# Supplementary material for: Unraveling the features of somatic transposition in the Drosophila intestine
Source: EMBO J. 2021 Feb 26;40(9):e106388. doi: 10.15252/embj.2020106388 (PMC8090852; doi:10.15252/embj.2020106388)
Supplement: Supplementary file 1 — Appendix [file EMBJ-40-e106388-s017.pdf]

Unraveling the features of somatic transposition in the *Drosophila* intestine

Katarzyna Siudeja*[1,2, #], Marius van den Beek*[1,2], Nick Riddiford[1,2], Benjamin Boumard[1,2], Annabelle Wurmser[1,2], Marine Stefanutti[1,2], Sonia Lameiras[3], Allison J. Bardin[1,2, #]

[1] Institut Curie, PSL Research University, CNRS UMR 3215, INSERM U934, Stem Cells and Tissue Homeostasis Group, Paris, France.
[2] Sorbonne Universités, UPMC Univ Paris 6, Paris, France
[3] ICGex Next-Generation Sequencing platform, Institut Curie, PSL Research University, 75005 Paris, France
* Equal contribution
# Authors for correspondence (katarzyna.siudeja@curie.fr, allison.bardin@curie.fr)

Appendix

Content:
Appendix Figure S1
Appendix Figure S2
Appendix Figure S3
Appendix Figure S4
Appendix Table S1

Appendix Figure S1

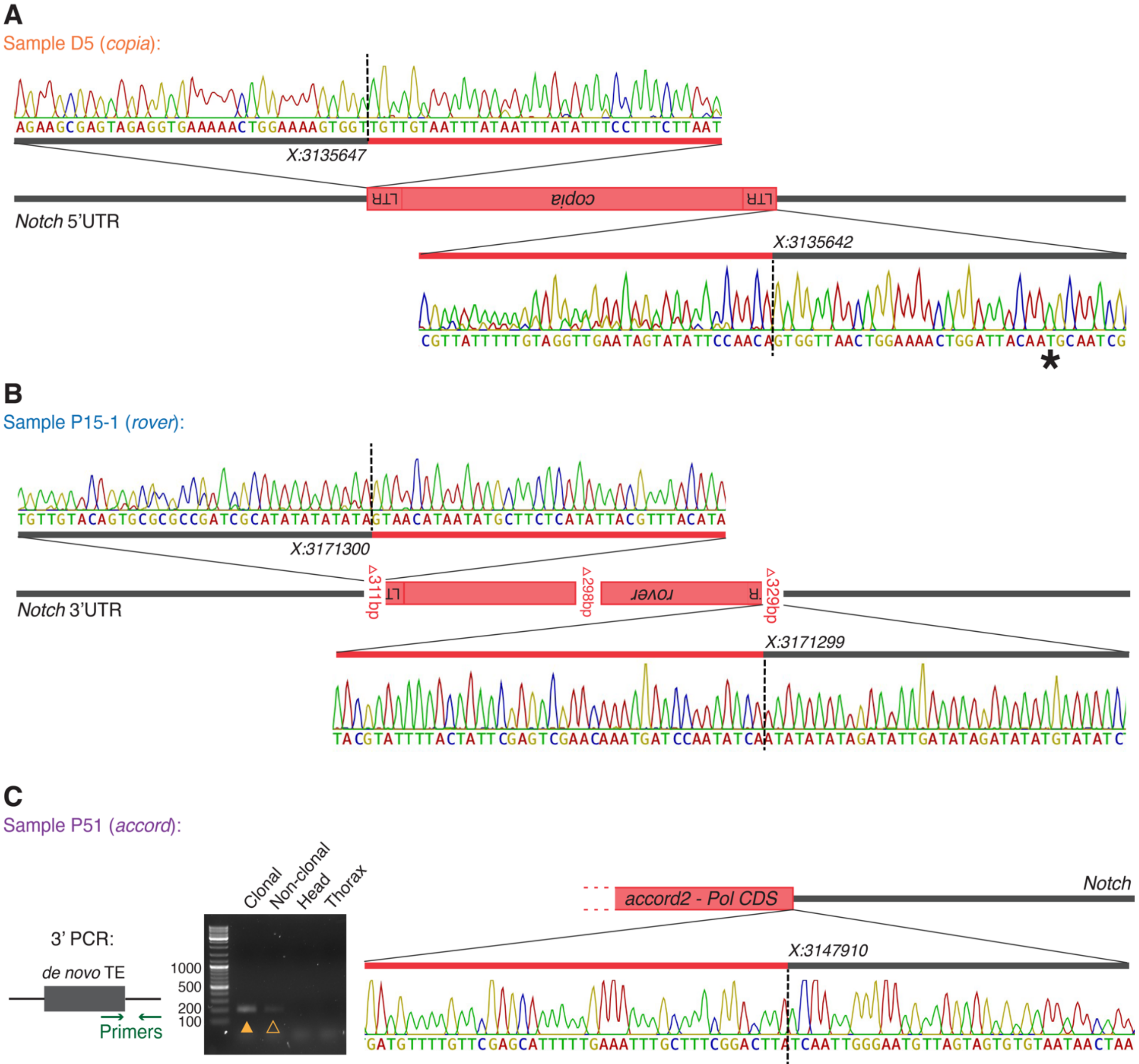

Appendix Figure S1 – Validation of the somatic TE insertions in *Notch* identified in the clonal gut samples.

Sequence validation of the TE inserts amplified from the clonal samples D5, P15 and P51. Original genomic sequences are depicted in black and TE fragments in red (based on the canonical TE sequences). Representative Sanger traces of sequences around insertion breakpoints are shown. Vertical dashed lines indicate insertion breakpoints. For A and B, agarose gels of the PCR amplicons are presented in Fig 1D.

A A full-length *copia* integration in the 5'UTR of *Notch* from sample D5. The TE is inserted in a reverse orientation and its entire length was sequence-verified. A star indicates the *Notch* translation initiation codon (ATG) 26bp downstream of the insertion.

B A truncated *rover* insertion in the 3'UTR of *Notch* from sample P15. Identified LTR and internal deletions relative to the *rover* canonical sequence are indicated.

C The 3’ breakpoint of the *accord2* insertion from sample P51. The breakpoint was PCR-amplified with primers inside and downstream of the TE insert. The yellow arrowhead indicates the expected 230bp amplificon detected in the clonal DNA. The same band was also detected in the non-clonal adjacent gut control, suggesting a contamination with clonal cells upon manual dissection. However, this amplicon was not present in the head and thorax controls isolated from the same fly. The amplicon was sequence verified by Sanger sequencing.

Appendix Figure S2

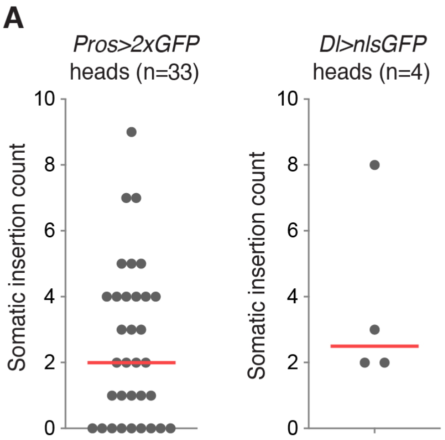

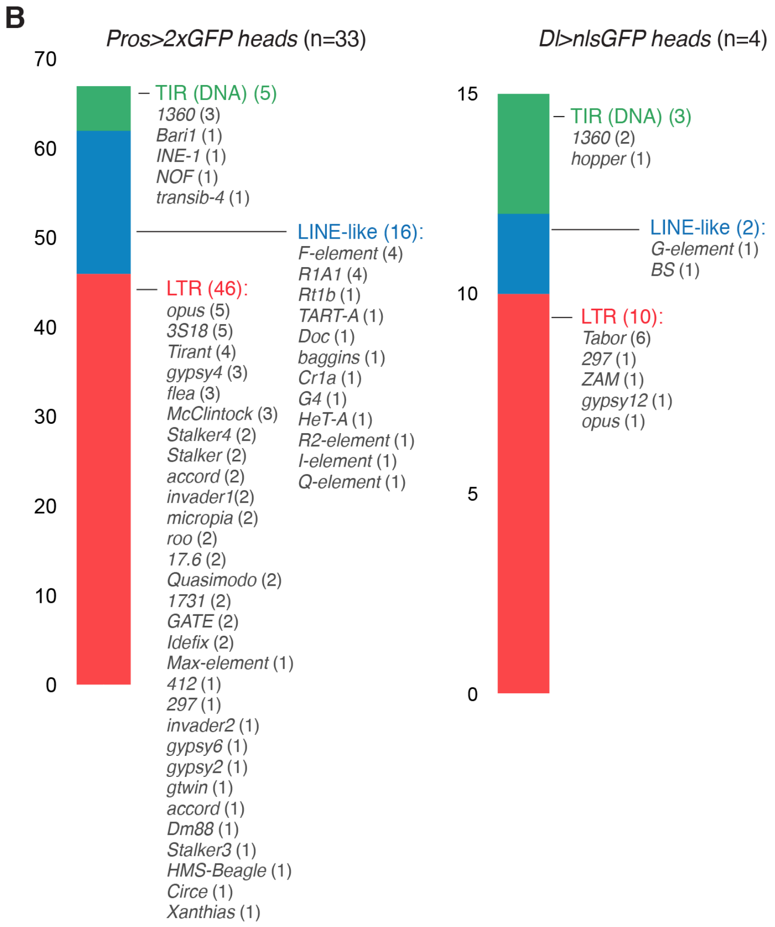

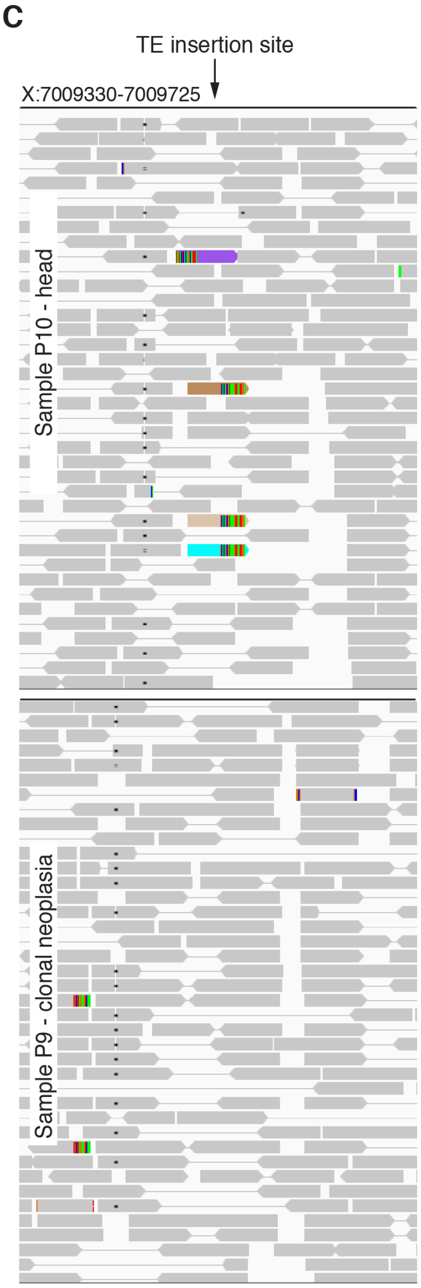

Appendix Figure S2 – Rare somatic transposition events are found in head samples

A The frequency of head-specific somatic insertion sites in the *Pros>2XGFP* and *Delta>nlsGFP* genetic backgrounds. Red lines represent median values.

B The distribution of TE classes active in the heads of two genetic backgrounds studied.

C The IGV screenshot of a head-specific TE insertion site from sample P10 (head) and its neoplasia control (sample P9). Bars represent sequencing reads. The reads supporting the TE insertion are colored.

Appendix Figure S3

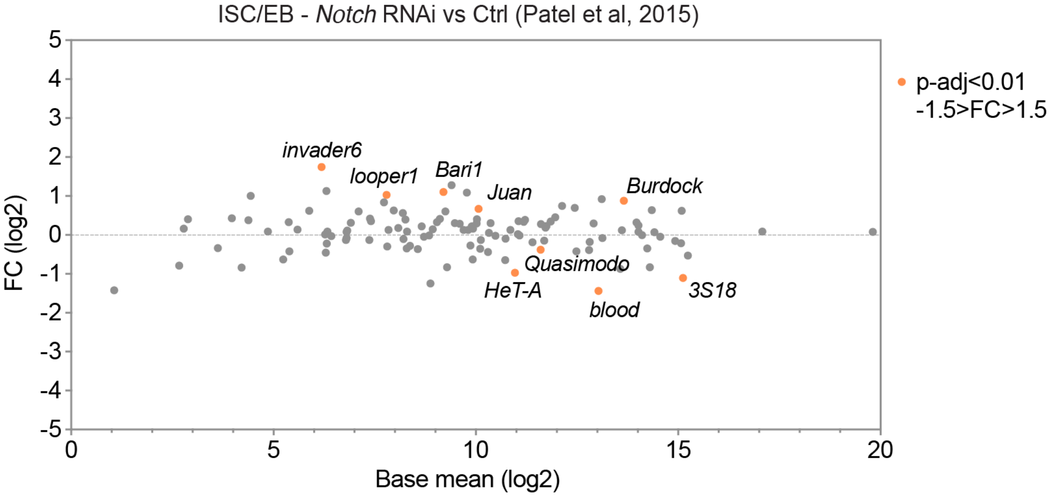

Appendix Figure S3 - *Notch* depletion in gut progenitor cells has a minor effect on TE expression levels.

Differential expression of TEs in FACS-sorted wild-type or *Notch*-depleted gut progenitor cells (*escargot* positive ISCs and enteroblasts). The data is from Patel et al, Nat Cell Biol, 2015. TEs with p(adj)<0.01 and -1.5>FC>1.5 were considered differentially expressed (labeled and marked in orange).

Appendix Figure S4

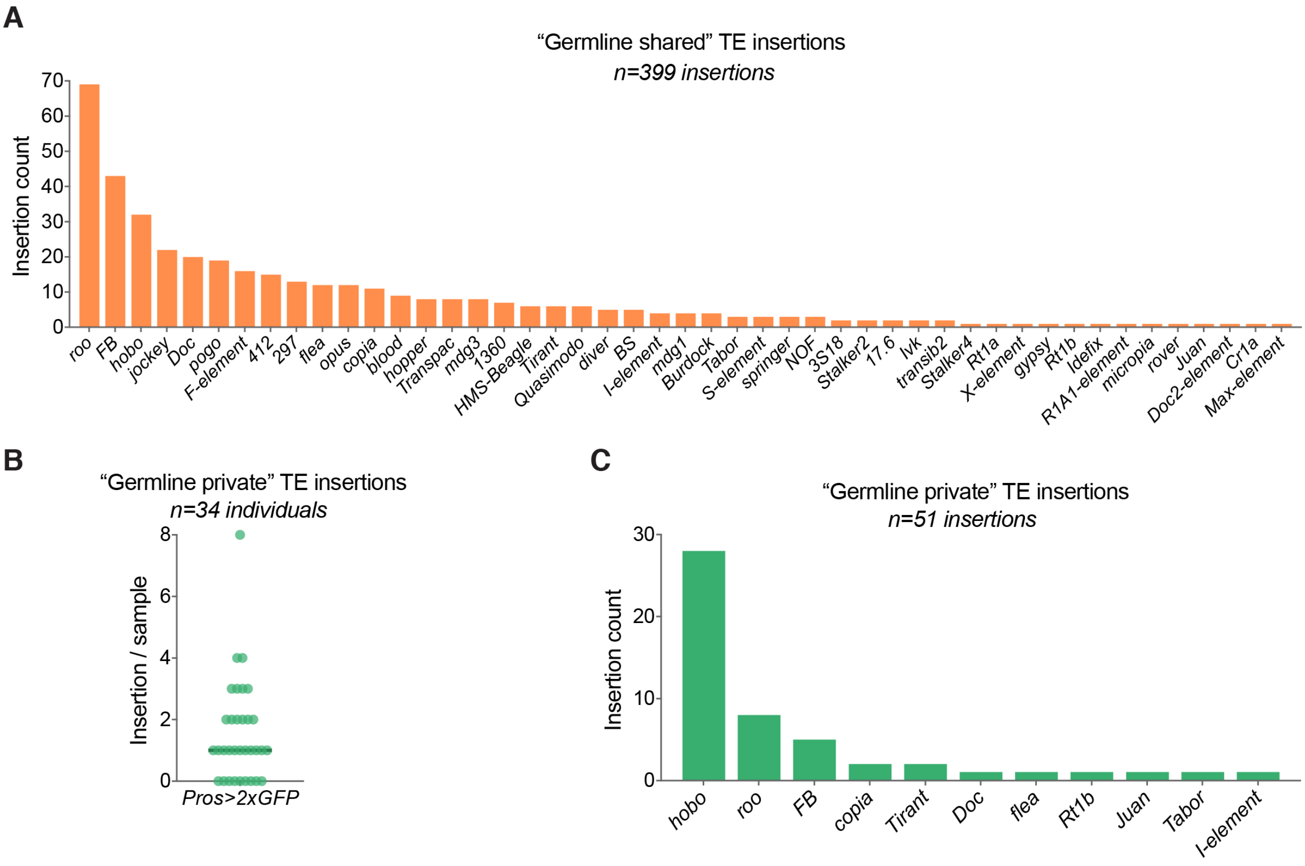

Appendix Figure S4 – Germline TE insertions in the *Pros>2xGFP* genetic background

A TE family distribution of “germline shared” non-reference TE insertions (with a TSD) present in all 34 *Pros>2xGFP* individuals sequenced. These insertions represent fixed germline variants.

B Frequencies of mapped “germline private” non-reference TE insertions (with a TSD), present in only a single individual (both in gut and head). Black line represents the mean value of 1.5 insertion/individual.

C TE family distribution of “germline private” insertions from B.

Appendix Table S1 - Primers used for PCR-validations of the *Notch* TE insertions (Fig.1 and Appendix Fig. S1)

Sample	Insertion	Forward primer	Revers primer
P15	*rover* (3'UTR)	CGCGCAAGGATAATTGGATGG	GCGTAGTCTTATGGCCTAGTG
P15	*rover* (Intron)	CCAGGCTGCAATTACTTTAATT	TGTAAAATGCAAGCGGAATGC
P47	*rover*	GCAGCATTTGGTCCAAACGTT	ATAAAATGCGCCACAAGACGAG
P51	*accord2*	TAGGTACGACCCAAACGGGTG	AACCGACACTTGTGCAGGAAG
D5	*copia*	GCATACTAAACCTAAACTCGCAGT	TTCTTTGGCAAGCGTTTTGGAA
