# Supplementary material for: Unraveling the features of somatic transposition in the Drosophila intestine
Source: EMBO J. 2021 Feb 26;40(9):e106388. doi: 10.15252/embj.2020106388 (PMC8090852; doi:10.15252/embj.2020106388)
Supplement: Supplementary file 2 — Expanded View Figures PDF [file EMBJ-40-e106388-s004.pdf]

Expanded View Figures

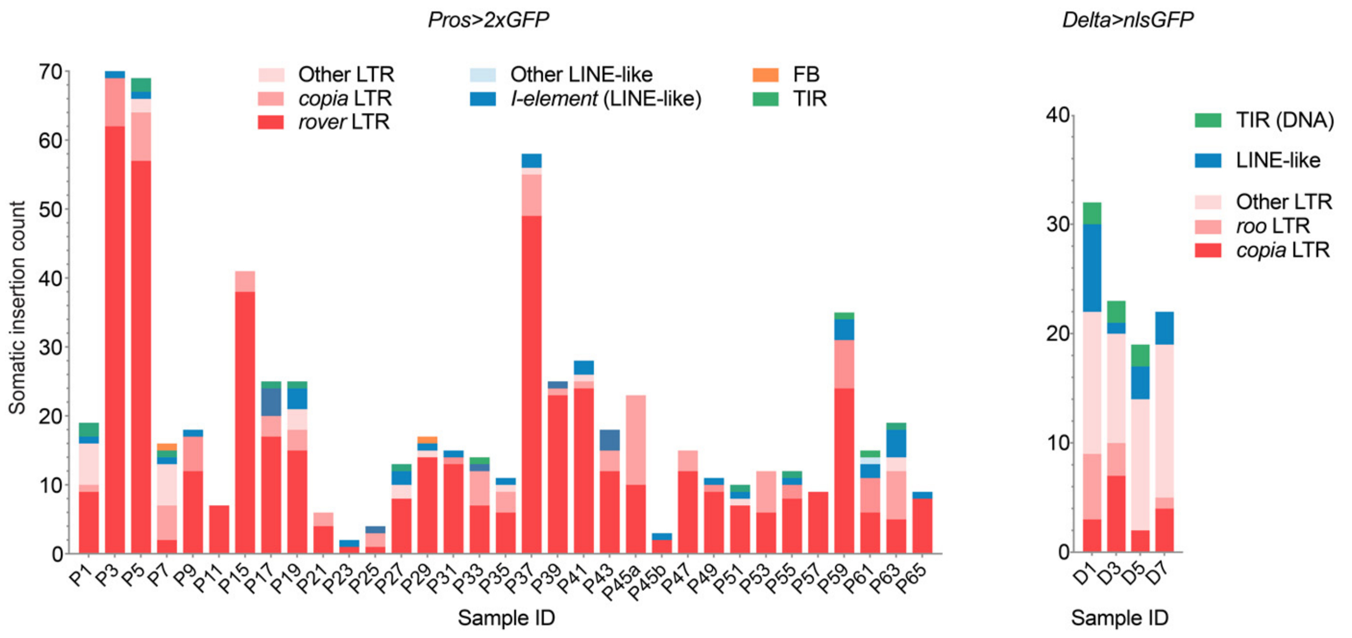

Figure EV1. Class distribution of mobile TE families in all clonal samples sequenced.

TEs were categorized in four main classes: LTR—long terminal repeat retrotransposons (in red), LINE-like—non-LTR retrotransposons (in blue), TIR—terminal inverted repeat DNA transposons (in green), and FB—foldback element (in orange). For each class, one or two most active families are highlighted with dark colors.

 © 2021 The Authors

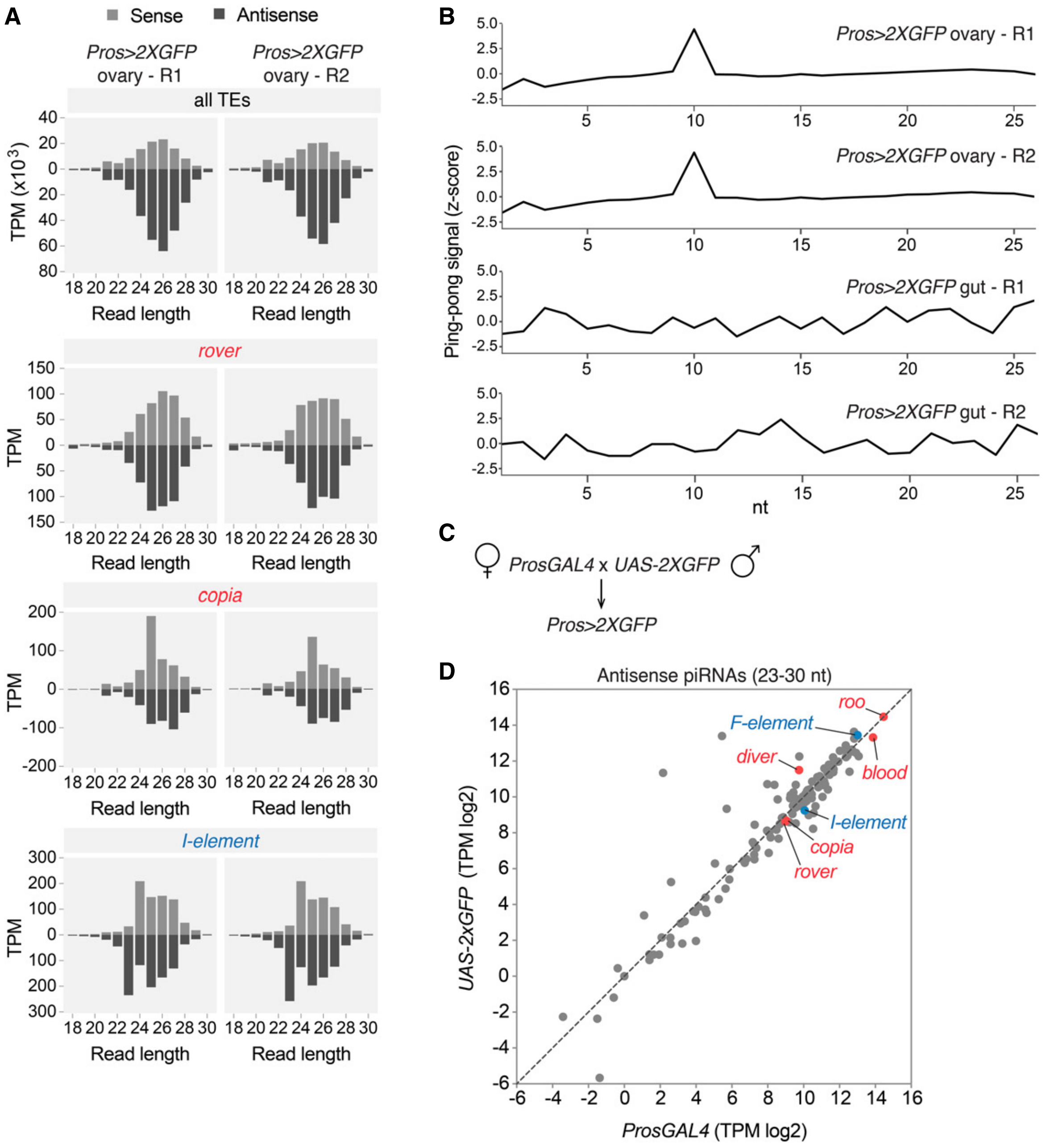

Figure EV2. The analysis of small RNA fractions isolated from ovaries.

A The size distribution of sense and antisense reads from *Pros > 2xGFP* ovary small RNA fractions mapping to all TEs (upper panel) or selected TE families mobilizing in the gut.

B The complementary sense and antisense read overlap (z-score) calculated on the 23–30 nt long small RNA populations from ovary and gut samples. The 10nt overlap detected in ovary, but not gut samples, is a signature of the piRNA "ping-pong" cycle.

C Parental fly crossing scheme used to obtain the *Pros > 2xGFP* genotype used in this study.

D Scatter plot showing normalized TE-mapping antisense piRNA levels from ovaries of two parental stocks used to obtain the *Pros > 2xGFP* flies. TEs generating most of the somatic gut insertions are highlighted in red (LTR elements) or blue (LINE-like).

Data information: In (A), R1 and R2 are two biological replicates.

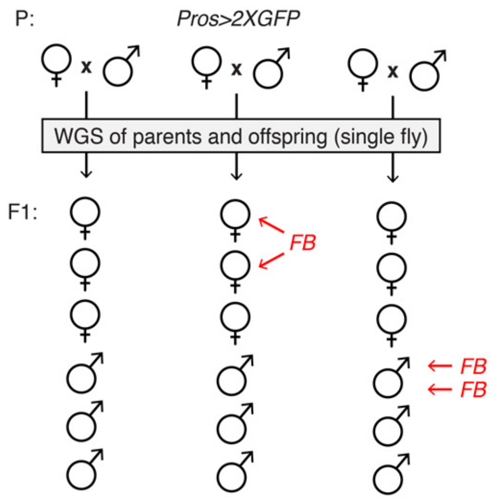

Figure EV3. The analysis of germline TE insertional activity.

Schematic representation of individual flies sequenced to detect germline TE insertions transmitted to the progeny (F1) of the *Pros* > *2xGFP* parents (P). Detected *de novo* germline TE insertions are indicated with horizontal red arrows. We detected three germline *de novo foldback* element insertions. Two of those were present in one male and one was detected in two sibling females. FB —*foldback* element.

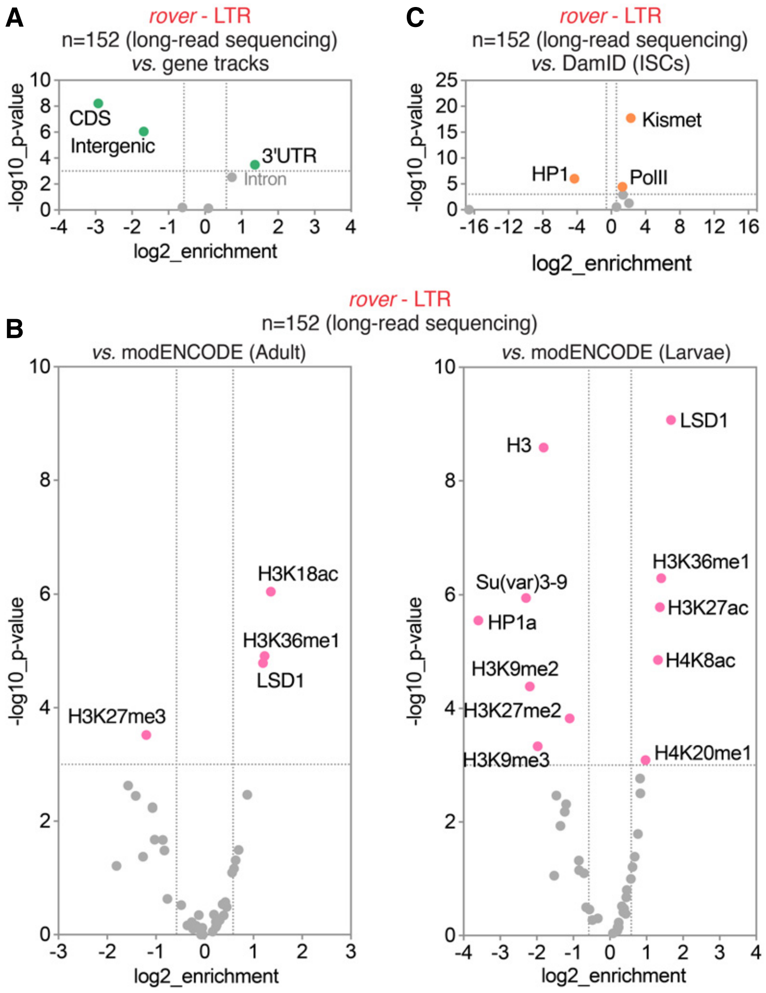

Figure EV4. Enrichments of putative somatic singleton insertions identified with the long-read sequencing of bulk gut DNA.

- A Candidate singleton insertion sites of *rover* LTR elements were depleted from intergenic and exonic sequences and enriched in 3'UTR regions of the fly genome.
- B Correlations of singleton insertion sites of *rover* elements with modENCODE tracks for adult fly (left) and larval (right) tissues.
- C Correlations of singleton insertion sites of *rover* elements with DamID tracks for adult fly intestinal stem cells (ISC).

Data information: Colored data points and labels highlight significant positive or negative correlations (Fisher's exact test with Benjamini–Hochberg correction, $P < 0.001$, $-1.5 >$ enrichment > 1.5).

 © 2021 The Authors

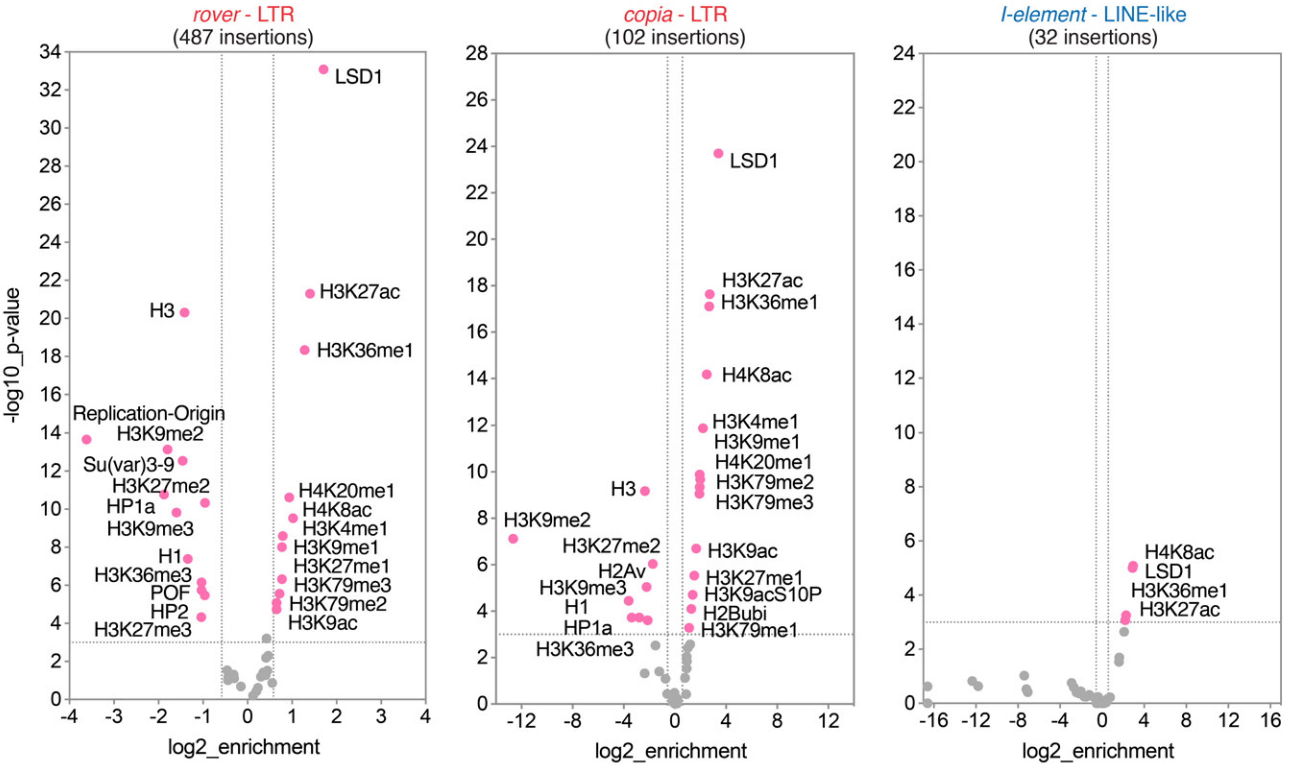

Figure EV5. Correlations of somatic insertion sites from the short-read sequencing of clonal samples with modENCODE tracks for *Drosophila* larvae.

Three most represented TE families (*rover*, *copia*, and *I-element*) are plotted. Colored data points and labels highlight significant positive or negative correlations (Fisher's exact test with Benjamini–Hochberg correction, $P < 0.001$, $-1.5 >$ enrichment > 1.5). Insertions from the *Pros* > *2xGFP* clonal gut short-read sequencing samples were used for all plots.
