# Supplementary figures and images for: Unraveling the features of somatic transposition in the Drosophila intestine
Source: EMBO J. 2021 Feb 26;40(9):e106388. doi: 10.15252/embj.2020106388 (PMC8090852; doi:10.15252/embj.2020106388)

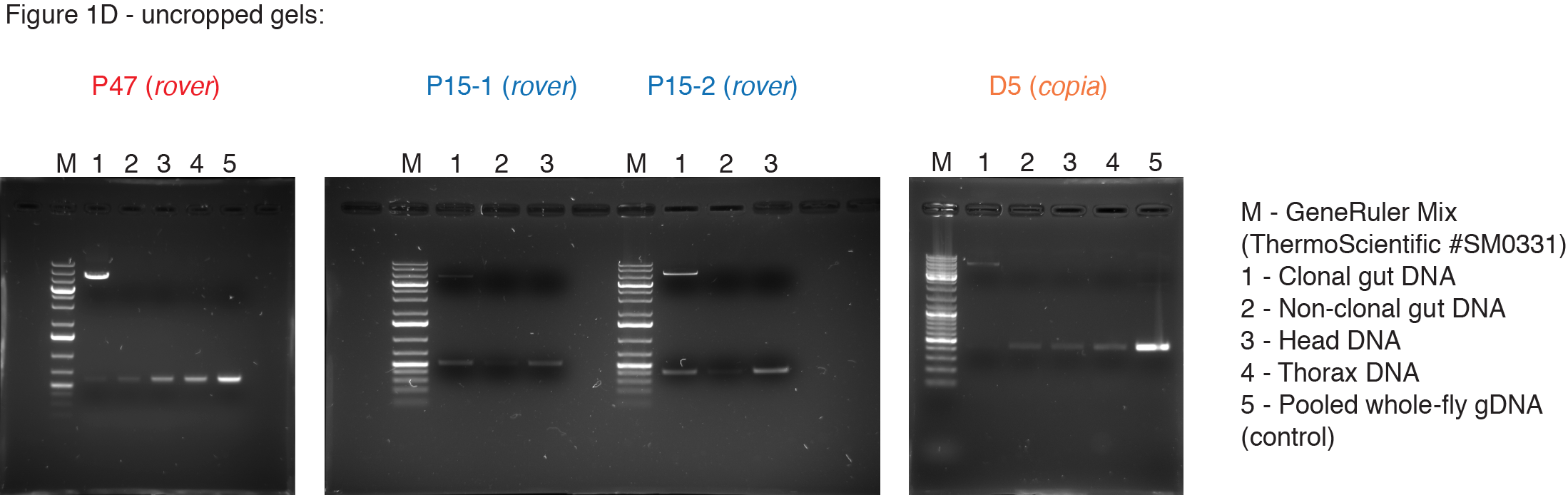

Supplement: Supplementary file 13 — Source Data for Figure 1D [file EMBJ-40-e106388-s008.png]
